# Effects of Portulaca oleracea ethanolic extract on reproductive system of aging female mice 

**Published:** 2016-03

**Authors:** Akram Ahangarpour, Zohreh Lamoochi, Hadi Fathi Moghaddam, Seyed Mohamad Taghi Mansouri

**Affiliations:** 1 *Health Research Institute, Diabetes Research Center, Department of Physiology, School of Medicine, Ahvaz Jundishapur University of Medical Sciences, Ahvaz, Iran.*; 2 *Physiology Research Center. Department of Physiology, Ahvaz Jundishapur University of Medical Science, Ahvaz, Iran. *; 3 *Department of Pharmacology, Physiology Research Center, Ahvaz Jundishapur University of Medical Sciences, Ahvaz, Iran.*

**Keywords:** *Aging*, *D-galactose*, *Portulaca oleracea L (Purslane)*, *antioxidant enzyme*, *sex hormones*

## Abstract

**Background:**

Aging contains morphological and functional deterioration in biological systems. D-galactose (D-gal) generates free radicals and accelerates aging. Portulaca oleracea (Purslane) may have protective effect against oxidative stress.

**Objective:**

Purslane ethanolic extract effects were evaluated on antioxidant indices and sex hormone in D-gal aging female mice.

**Materials and Methods:**

48 female NMRI mice (25-35 gr) were randomly divided into, 6 groups: 1- control (normal saline for 45 days), 2- Purslane (200 mg/kg for last 3 weeks), 3-D-gal (500 mg/kg for 45 days), 4-D-gal+Purslane, 5- Aging, 6-Aging+Purslane. Sex hormones, antioxidants and malondialdehyde (MDA) level of ovary and uterus were measured. Histological assessment was also done.

**Results:**

In D-gal treated and aging animals, LH and FSH levels were significantly increased (p<0.001) while estrogen and progesterone levels were significantly reduced (p<0.001) in comparison with control group. MDA contents were significantly increased in ovaries and uterus of D-gal and aging groups (p<0.01). Superoxide dismutase (SOD) (p<0.001) and catalase (p<0.01) activities were significantly decreased in both aging and D-gal treated animals. Ovarian follicles were degenerated and atrophy on uterine wall and endometrial glands was observed in D-gal and aging groups. Alteration in hormone levels, MDA contents and antioxidant activity were significantly reversed by Purslane (p<0.05). Purslane could also improve histological changes such as atrophy of endometrium.

**Conclusion:**

These findings indicate that Purslane can attenuate aging alternations induced by D-gal and aging in female reproductive system.

## Introduction

Aging contains a lot of morphological and functional deterioration in biological systems which associate with various molecular, cellular and organic changes (1). Aging is related to progressive decrease in immune activity or immunosenescence (2). In fact, aging is produced by endogenous oxygen radical accumulation from oxidative modification of biomolecules (lipids, proteins and nucleic acids) (3). It has been observed that ovary is the first major organ weakened in aging female mice (4). 

In rodents, feedback of gonadal sex steroid hormones in hypothalamus and pituitary gland were stopped during process of reproductive aging (5). Malondialdehyde (MDA) as cytotoxic product of cells, is obtained from lipid peroxidation damage and necrosis (6, 7). Reactive oxygen species (ROS) are involved in more than 100 diseases. ROS and antioxidants are in balance in healthy body. When balance is disturbed in direction of ROS excess, oxidative stress (OS) happens. Antioxidants neutralize free radicals and as scavengers are operating. They have created considerable interest in overcoming opposed and pathological results of OS and this pathological effect of OS may be achieved by decreasing of output ROS or increasing quantity of available antioxidants (8). Enzymatic and non- enzymatic antioxidants in cells preserve against oxidative damage (9). 

Some of endogenous antioxidant enzymes, for example superoxide dismutase (SOD) and catalase (CAT) are important in this process (10). The D-gal, reducing sugar, is in body. It responds with free amines of amino acids in peptides to create advanced glycation end products (AGE), which bring about activation of receptor for advanced glycation end products (RAGE). This series of biochemical events, results an OS and cellular damage. D-gal has a first role in pathogenesis of aging (11). D-gal has been injected into mice or rats for pharmacological researches (12). D-gal allowed body cells to generate excess free radicals, precipitated aging, and can lead to premature ovarian failure (13). Portulaca oleracea L. (Purslane) is a grassy plant with small yellow flowers and stems, sometimes flushed red or purple, which grows over world wide area (14). 

It used as a potherb in Asian, central European and Mediterranean countries, which is utilized as one of medicinal plants and has been given term “Global Panacea” (15). Recent pharmacological research has shown many capabilities of Purslane like muscle relaxant activity, decrease in locomotor activity, increased in onset time of pentylenetetrazole-induced convulsion, analgesic, anti-inflammatory effects and antioxidant properties (16). Purslane contains many compounds, such as alkaloids, omega-3 fatty acids, coumarins, flavonoids, polysaccharide, cardiac glycosides, and anthraquinone glycosides (17). In a literature review, there was no evidence of hepatic, renal or lung toxicity in animal exposed to Purslane (18-20).

Purslane also has been introduced as major source of antioxidant vitamins such as α-tocopherol, ascorbic acid, β-carotene, also glutathione. Due to its nutritive and antioxidant properties, it has qualified as unique food of future (21). It is remarkable that no sign of notable toxicity of Purslane has been reported yet (22). Estrous cycle of mice consisted of endocrine, behavioral and physiological events, which occurs every 4-6 days during reproductive life span unless terminated by pregnancy, pseudopregnancy or anestrous. For facility only 4 stages of estrous cycle are proestrous, estrus, metestrus and diestrus. Examining smears of cells from vagina define stage of estrus cycle (23). 

Ethanol extract of Purslane has been reported to have an estrogenic activity, which might be due to presence of flavonoids, which operate estrogenic activities in rat (24). 

Since aging in societies is growing and there are few research documents about aging model and effects of medicinal plants such as Purslane on this model and its reproductive complications, present study attempted to investigate beneficial effects of Portulaca oleracea ethanolic extract on the reproductive system of aging female in a mouse model. 

## Materials and methods

**Plant material**

Purslane has been collected from Ahvaz suburb gardens and have identified by Faculty of Pharmacy (A14229001P) Ahvaz Jundishapur University of Medical Sciences herbarium. This study was approved by the institution's Animal Ethics Committee (APRC-93-80). 

In order to prepare alcoholic extracts of Purslane, standard methods of extraction were used. Aerial parts including stem and leaves of Purslane were collected and dried correctly then powdered using a mixer. 50 gr of dry powder was added to 450 ml ethanol 70% (30 water/ 70 ethanol) and stored 72 hr at room temperature to absorb moisture well. The mixture was filtered and centrifuged for 10 min at 3000 RPM (25, 26). After evaporation of alcohol, supernatant was dried at 37^o^C and weight of obtaining from extract was measured as 9.5% compared to dry plant and kept at -4^o^C until use.

**Animals**

Two categories of female NMRI mice (n=8 in each group) which were obtained from Ahvaz Jundishapur University of Medical Sciences (AJUMS) animal house have been used in this experimental study; 32 young (3 months old) and 16 old animals (18-24 months old) (27). The mice were housed and kept under standard laboratory conditions (12 hr dark/light cycle, relative humidity of 50±5% and at a temperature of 22±3^o^C). Animals had ad libitum access to commercial food (pellet) and water throughout the experiment. 

**Drugs and Treatment Schedule**

D-gal solution and standardized ethanolic extract of Purslane were used. D-gal was dissolved in saline for subcutaneous (SC) administration and prepared according to required concentration 500 mg/kg daily for 45 days (21, 28). Purslane extract was dissolved in saline and was given 200 mg/kg by gavage method (21, 25). Young groups divided into 4 subgroups as below:

1) Control group initially received 0.1 ml normal saline daily for 24 days by SC and was followed by concomitant administration of 0.1 ml normal saline for 21 days by gavage. 

2) Purslane group initially received 0.1ml normal saline daily for 24 days by SC and was followed by concomitant administration of 200 mg/kg (25) Purslane for 21 days by gavage. 

3) D-gal group initially received 500 mg/kg D-gal daily for 24 days and was followed by concomitant administration of 0.1 ml normal saline for 21 days by gavage. 

4) D-gal + Purslane group initially received 500 mg/kg D-gal daily for 24 days and was followed by concomitant administration of 200 mg/kg Purslane for 21 days by gavage. 

Old groups were divided into two subgroups as below:

5) Aging group initially received 0.1 ml normal saline daily for 24 days by SC and was followed by concomitant administration of 0.1 ml normal saline for 21 days by gavage. 

6) Aging + Purslane group initially received 0.1ml normal saline daily for 24 days and was followed by concomitant administration of 200 mg/kg Purslane for 21 days by gavage. 

First 0.2 ml solution containing 100 µg of estradiol valerate, dissolved in olive oil, was injected intramuscularly. After 42 hr, 50 mg of progesterone administrated intramuscularly and 6 hr later on, vaginal smear was prepared, for checking mice estrus cycle (25). Stages of estrus cycles were determined in last 4 days of study (23). Smears on slides were fixed and stained with 1% methylene blue. Slides were reviewed using light microscopy. 

At the end of, the mice were weighed and anaesthetized with Ketamine/Xylazine. Blood samples were collected and centrifuged from heart. The obtained serum was collecte and kept on -20^o^C for hormonal assessments. Ovaries and uterus of each animal were removed and organ weight/ body weight ratio was calculated. Small pieces of uterus and left ovary were hemogenized for assessment of MDA, SOD and CAT. Other pieces of uterus and right ovary were fixed in 10% formalin for histological studies.

**Histology assessments**

Formalin fixed samples were embedded in paraffin, sectioned (5 μm) and stained with haematoxylin and eosin (H&E), as previously described (29, 30). Histopathologic examination was performed by light microscopy. H&E stained slides were evaluated for signs of histological changes such as follicular degeneration and endometrial atrophy.

**Experimental measurement**

**Hormonal assessment**

Mouse luteinizing hormone (LH) and follicle-stimulating hormone (FSH) concentrations in serum samples were measured by ELISA assays kits (CUSABIO China Inc). Minimum detectable dose of mouse LH and FSH is typically ˂0.5 and 0.35 mlU/ml, respectively. Sensitivity of this assay, or lower limit of detection was defined as lowest mouse LH and FSH concentration that could be differentiated from zero.

This assay has high sensitivity and excellent specificity for detection of mouse LH and FSH. No significant cross-reactivity or interference between mouse LH and FSH and analogues were observed. For direct quantitative determination of progesterone and estrogen levels in serum, samples were measured by ELISA assays kits (Diagnostics Biochem Canada Inc). Sensitivity progesterone and estrogen are 0.1 ng/ml and 10 pg/ml, respectively.

**MDA, SOD and CAT assessment in the uterus and ovarian tissue**

To measured antioxidant and MDA, left ovaries and uterus of all animals were homogenized and centrifuged using 5000 RPM. Supernatant was collected gently and kept in -20^o^C until enzyme measurements. MDA, SOD and CAT ELISA kits have been obtained from Biocore Diagnostik Ulm GmbH, Germany and measurement was done according to manufacturer's instructions. MDA assessment in uterus and ovary tissues was measured by ELISA assay kit that detects MDA levels colorimetrically, in range of 0.78-50 µM with 0.1 µM sensitivity, intra and inter assay coefficient of variation 5.8% and 7.6%, respectively.

**Antioxidant enzyme activities**

SOD assessment in uterus and ovary tissue was measured by ELISA assays kits. SOD activity determination in range of 5-100 U/ml, 1 U/ml sensitivity, intra and inter assay coefficient of variation 5.8% and 7.2% respectively. CAT assessment in uterus and ovarian tissue was measured by ELISA assays kits. CAT activity determination in range of 1-100 U/ml, 0.5 U/ml sensitivity, intra and inter assay coefficient of variation 6.3% and 7.9%, respectively.

**Statistical analysis**

All results were expressed as means±SEM and differences were considered one-way ANOVA followed by post hoc LSD test to evaluate differences between different groups. P <0.05 was considered significant [SPSS software (Statistical Package for the Social Sciences, version 15.0, SPSS Inc, Chicago, Illinois, USA].

## Results

**Effects of ethanolic extract of Purslane on antioxidant indices of the ovarian and uterine tissues**

**Figure 1:**
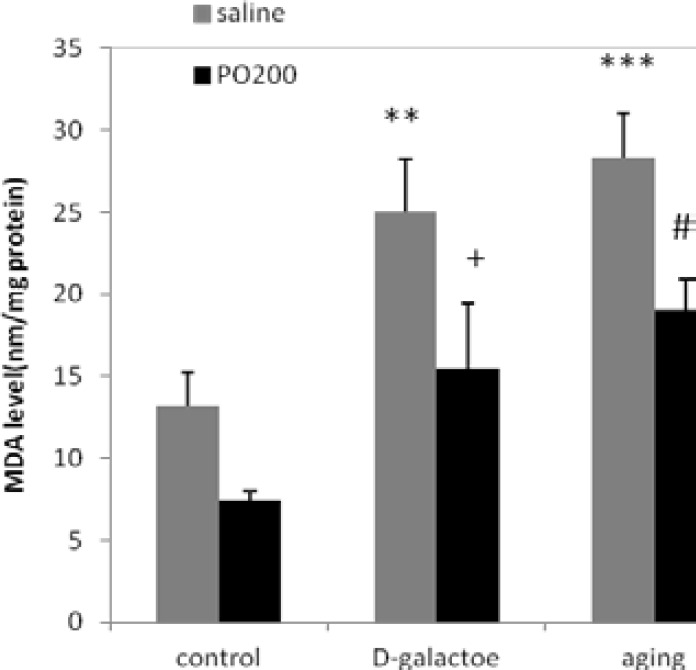
Effect of ethanolic extract of Purslane (PO) on MDA level in ovary and uterus in normal and aging mice model; n=12; Mean±SEM. One-way ANOVA and post hoc LSD tests; **p<0.01, ***p<0.001 vs. Control group, #p<0.05 vs. Aging group, + p<0.05 vs D-gal group

**Figure 2:**
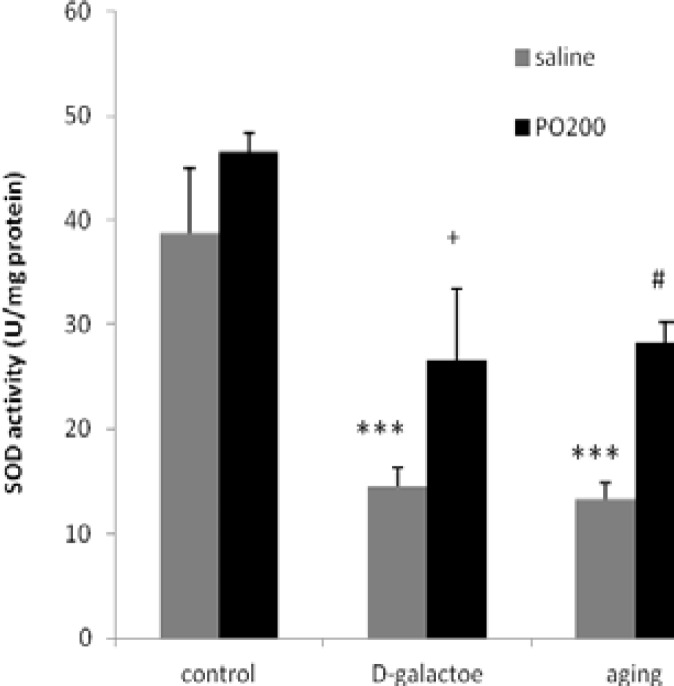
Effect of ethanolic extract of Purslane (PO) on SOD activity in ovary and uterus in normal and aging mice model; n=12; Mean±SEM. One-way ANOVA and post hoc LSD tests; *** p<0.001 vs. Control group, #p<0.05 vs. Aging group, + p<0.05 vs D-gal group.

**Figure 3:**
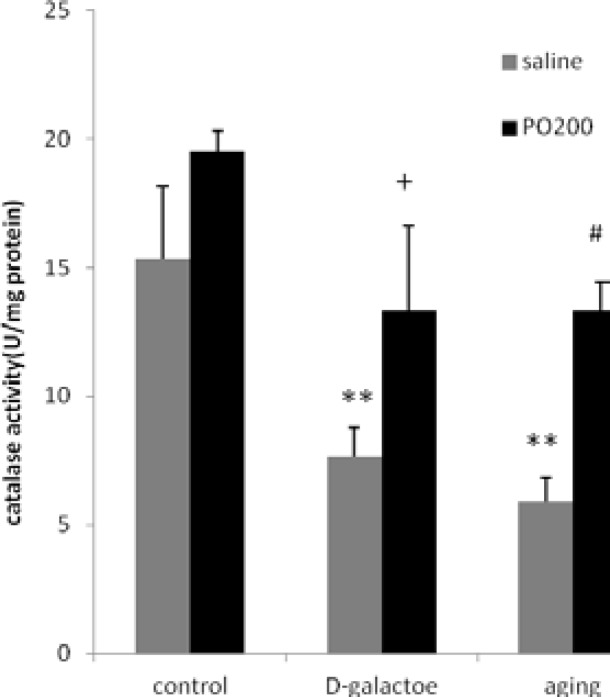
Effect of ethanolic extract of Purslane (PO) on CAT activity in ovary and uterus in normal and aging mice model; n=12; Mean±SEM. One-way ANOVA and post hoc LSD tests; **p<0.01 vs. Control group, #p<0.05 vs Aging group, + p<0.05 vs D-gal group.

Values and statistical comparisons of MDA level are shown in figure 1. SOD and CAT activities in experimental groups are shown in figure 2-3, respectively. In this study, it was shown that D-gal interrupted estrous cycle somehow, by alteration MDA, SOD and CAT. MDA level (nmol/ mg protein) in ovary and uterus, increased in aging (p<0.001) and D-gal groups (p<0.01) compared to control group. 

SOD and CAT enzyme activities (U/mg protein) in ovary and uterus were decreased in D-gal and aging groups compared to control group (p<0.001 and p<0.01, respectively). MDA level (nmol/ mg protein) in ovaries and uterus of D-gal+Purslane and aging+Purslane groups (p<0.05) were reduced significantly in comparison with aging and aging induced by D-gal groups. SOD and CAT (p<0.05) enzyme activity in normal treated mice with D-gal+Purslane and aging+Purslane groups were increased in comparison with D-gal and aging group. 

**Effects of ethanolic extract of Purslane on histology of the ovarian and uterine**

Control group ovaries contained graafian follicle with clear zona pelucida. Histological architecture of Purslane treated ovaries was similar to controls. In D-gal treated ovaries, degenerative changes was observed in follicles. 

**Figure 4:**
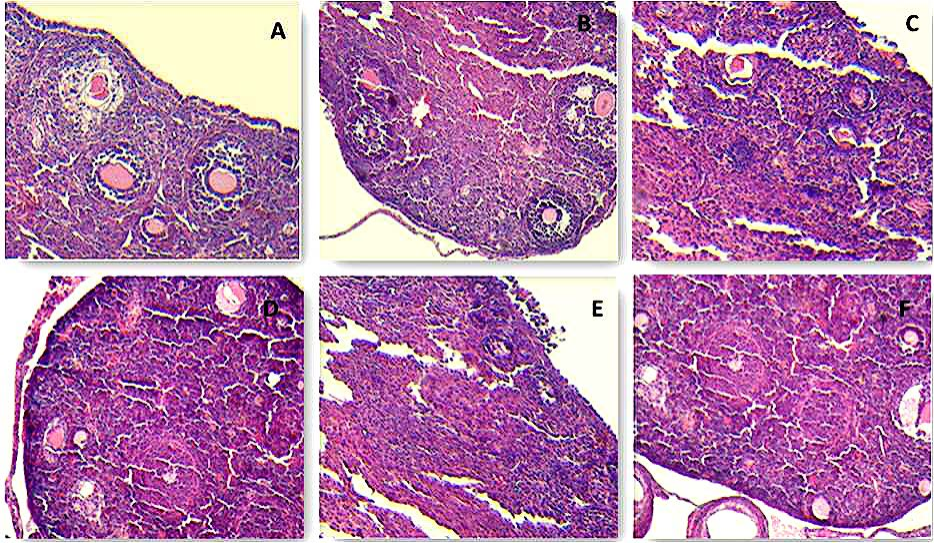
Photomicrographs of ovarian tissues. Effects of Purslane ethanolic extract on ovarian in aging mouse model. (A) control group, (B) Purslane control group; (C) D-gal group, (D) D-gal+Purslane group (E) Aging group (F) Aging+Purslane group (H&E staining, 400×).

**Figure 5.**
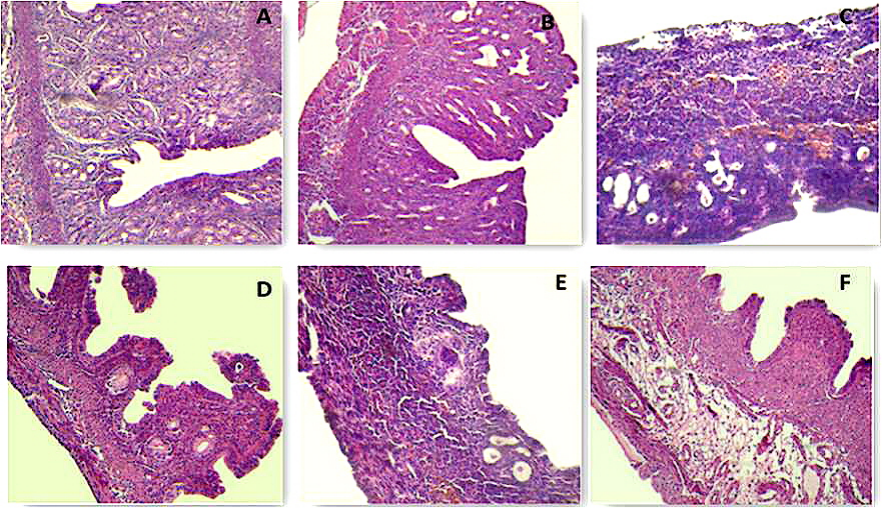
Photomicrographs of uterine tissues. Effects of Purslane ethanolic extract on Ovarian in aging mice model. (A) Control group; (B) Purslane control group; (C) D-gal group, (D) D-gal+Purslane group; (E) Aging group; (F) Aging+Purslane group (H&E staining; Magnifications: C is 250× and other figures are 100×).

Graafian follicle and zona pellucida were also not observed. Nucleus of oocytes could also not be defined. In aging group, degeneration of follicles in different stages was observed. Purslane could significntly reverse histological changes in ovaries of both D-gal and aging groups (Figure 4). Normal histological architecture was observed in both control and Purslane groups uterus. Endometrium was atrophic and a few small glands was observed in D-gal and aging groups. These alterations were significntly improved by Purslane (Figure 5). 

**Effect of Purslane on serum levels of sex hormones**

Analysis effect of Purslane on sex hormones (Table II) shows that the LH level was significantly increased in D-gal and aging compared with control group (p<0.001). Significant decreased has been shown that LH level in D-gal +Purslane and aging +Purslane groups treated animals compared to D-gal and aging groups (p<0.05). FSH level was significantly increased in D-gal and aging groups compared to the control group (p<0.001). 

Purslane didnot change FSH level in any experimental groups. Estrogen and progesterone were also significantly decreased in D-gal and aging in comparison with control group (p<0.001). Purslane also significantly increased in D-gal+Purslane and Aging+Purslane groups in comparison with D-gal and aging groups (p<0.01, p<0.05 respectively). 

**Effects of ethanolic extract of Purslane on ovarian and uterine weight**

Also, administration of Purslane extract did not change the body weight, or percentage of ovarian and uterine weight in total groups (Table III).

**Table I:** Effects of ethanolic extract of Purslane on antioxidant indices in aging mice induced by D-gal

**Groups**	**MDA (nm /mg protein)**	**SOD (U/ mg protein)**	**CAT (U/ mg protein)**
Control	13.16 ± 2.10	38.72 ± 6.20	15.35 ± 2.79
Purslane (200 mg/kg)	7.43 ± 0.59	46.50 ± 1.85	19.49 ± 0.84
D-gal	25.07 ± 3.15**	14.55 ± 1.80***	7.64 ± 1.16***
D-gal+Purslane (200 mg/kg)	15.45 ± 3.99+	26.64 ± 6.83+	13.30 ± 3.35+
Aging	28.34 ± 2.71***	13.37 ± 1.52***	5.92 ± 0.91***
Aging+Purslane (200 mg/kg)	19.05 ± 1.85$	28.30 ± 2.01$	13.34 ± 1.10$

Data are presented as Mean±SEM.

** p<0.01,

*** p<0.001 vs. Control group,

+ p<0.05 vs. D-gal Group,

$ p< 0.05 as compared to aging group.

**Table II:** Effects of ethanolic extract of Purslane on sex hormones in aging mice induced by D-gal.

**Groups **	**LH (mlU/ml)**	**FSH (mlU/ml)**	**Estrogen (pg/ml)**	**Progesterone (ng/ml)**
Control	12.08 ± 1.46	85.51 ± 0.81	153.64 ± 2.35	10.39 ± 0.59
Purslane (200 mg/kg)	12.50 ± 0.41	87.74 ± 1.85	156.50 ± 2.83	10.47 ± 0.25
D-gal	23.44 ± 1.23***	96.60 ± 1.25***	113.76 ± 1.89***	6.15 ± 0.75***
D-gal+Purslane (200 mg/kg)	19.77 ± 0.27***+	99.09 ± 0.56***	124.85 ± 3.49***++	8.83 ± 0.97++
Aging	32.03 ± 0.74***	115.91 ± 2.06***	118.87 ± 2.65***	5.61 ± 0.85***
Aging+Purslane (200 mg/kg)	27.59 ± 0.48***$	116.37 ± 2.92***	128.14 ± 3.51***$	7.86 ± 0.32**$

Data are presented as Mean±SEM.

*** p<0.001 vs. Control group,

+ p<0.05,

++ p<0.01, vs. D-gal Group,

$ p<0.05, as compared with aging group.

**Table III:** Effects of ethanolic extract of Purslane on ovarian and uterine weight in aging mice induced by D-gal

**Groups**	**Body Weight (gr)**	**Ovarian and Uterine weight (gr)**	**%Ovarian and Uterine weight/B.W.**
Control	30.21 ± 1.24	0.15 ± 0.01	0.534 ± 0.05
Purslane (200 mg/kg)	31.46 ± 1.19	0.15 ± 0.02	0.494 ± 0.09
D-gal	30.11 ± 0.89	0.12 ± 0.01	0.428 ± 0.05
D-gal+Purslane (200 mg/kg)	31.41 ± 1.16	0.16 ± 0.02	0.570 ± 0.08
Aging	32.97 ± 0.77	0.13 ± 0.01	0.417 ± 0.02
Aging+ Purslane (200 mg/kg)	31.96 ± 1.22	0.13 ± 0.01	0.407 ± 0.02

Data are presented as Mean ± SEM.

## Discussion

Present study demonstrates that Purslane can significantly attenuate D-gal induced female mice reproductive age. It is well known that D-gal induces aging alterations similar to norml aging processes (31). As it was shown in results, LH and FSH levels were significantly increased in aging and D-gal treated animals. Ahangarpour *et al* previously showed that D-gal could increase LH and FSH levels in D-gal induced aging mouse model (32). Elevated basal FSH (and then LH) can be accounted reproductive and endocrinology signs of aging (33). Purslane did not change the FSH level in any experimental groups. 

Hosseini *et al* showed that hydroalcoholic (70%) extract (200 mg/kg) of Purslane had no significant effect on LH, FSH, estradiol and progesterone levels in the serum of normal mice (25). Administration of Purslane increased progesterone and estrogen levels in D-gal and aging mice. These alterations in hormone levels can explain disruption of estrous cycles in D-gal group. In mammals, aging of reproductive system, can reduce hypothalamic-pituitary-gonadal axis capacity (5). 

Chakraborty *et al* reported that reproductive aging in women with major changes in ovarian function is result of sudden loss of estrogen and progesterone secretion (34). Along with hormonal changes, histological changes were made. Endometrial atrophy and degeneration of ovarian follicles were accurred. D-gal administration caused significant increase in MDA content and significant decrease in antioxidant enzyme activities. It is revealed that excessive formation of ROS induced by D-gal increases aging in mice (1, 3, 4, 7). Sun *et al* have demonstrated that D-gal can significantly increase MDA content and induce significant decrease in serum estrogen and SOD activity in mice (13). 

Similar changes of normal aging animals have been reported by Yu *et al*) 35). Liu *et al* have reported that reproductive aging has been implicated by OS (36). Somatic aging in general, is involved in reproductive aging due to OS (35). OS causes damage to oocyte and embryos. Thus, use of antioxidant probably suppresses reproductive aging in females (37). In this study, administration of ethanolic extract of Purslane induced a significant decrease in MDA content while SOD and CAT enzymes activities were increased.

The exact mechanism of anti-aging effects of Purslane is not obtained from this study. Purslane contains isoflavones and bioflavonoids such as quercetin, kaempferol and myricetin anti-aging effects of these flavonoids have been reported (25, 38, 39). Dkhil *et al* demonstrated that Purslane increased antioxidant enzyme activities in hepatic, renal and testicular rat cells (21(. Protective effect of aqueous and ethanolic extracts of Purslane against generation of ROS and antioxidant defense in renal tissue and blood cells of rats was shown by Karimi *et al* (16, 40). 

## Conclusion

In conclusion, our results demonstrated that Purslane could significantly reduce MDA content, increase antioxidant enzyme activities (SOD and CAT) and attenuate female sex hormones in aging mice. Purslane could also reverse histoligical changes in these animals. Extrapolation of these results is not appropriate to human. However, these findings do provide stimulus for clinical studies.

## Note

***This article extracted from M.Sc. thesis. (Zohreh Lamoochi)***
